# Awareness of Human Papillomavirus (HPV) and HPV Vaccination amongst the General Population in Germany: Lack of Awareness and Need for Action

**DOI:** 10.1159/000525697

**Published:** 2022-07-18

**Authors:** Shachi Jenny Sharma, Volker H. Schartinger, Nora Wuerdemann, Christine Langer, Kathrin Möllenhoff, Lisa Collin, Liam Sutton, David Riedl, Alexander Kreuter, Matt Lechner, Ulrike Wieland, Jens Peter Klussmann

**Affiliations:** ^a^Department of Otorhinolaryngology, Head and Neck Surgery, Medical Faculty, University of Cologne, Cologne, Germany; ^b^Faculty of Medicine and University Hospital Cologne, Center for Molecular Medicine Cologne (CMMC), University of Cologne, Cologne, Germany; ^c^Department of Otorhinolaryngology, Medical University of Innsbruck, Innsbruck, Austria; ^d^Department of Otorhinolaryngology, Head and Neck Surgery, University of Giessen, Giessen, Germany; ^e^Institute of Medical Statistics and Computational Biology, Faculty of Medicine, University of Cologne, Cologne, Germany; ^f^Royal London Hospital, Barts Health NHS Trust, London, UK; ^g^UCL Cancer Institute, University College London, London, UK; ^h^Department of Medical Psychology, Medical University of Innsbruck, Innsbruck, Austria; ^i^Department of Dermatology, Venereology, and Allergology, Helios St. Elisabeth Hospital Oberhausen, University Witten-Herdecke, Witten, Germany; ^j^Institute of Virology, National Reference Center for Papilloma- and Polyomaviruses, University of Cologne, Faculty of Medicine and University Hospital of Cologne, Cologne, Germany

**Keywords:** Oropharyngeal squamous cell carcinoma, OPC, Human papillomavirus, Awareness rates, Germany, Europe

## Abstract

**Introduction:**

The oncogenic human papillomaviruses (HPV) types 16 and 18 contribute to more than 73% cases of all HPV-related cancers and commonly affect the anogenital and head and neck region, with rapidly rising incidence rates of HPV-related oropharyngeal squamous cell carcinomas (OPSCC). HPV vaccination has the potential to decrease the burden of HPV-related disease, but vaccination rates remain low in many countries. We investigated the level of awareness of HPV, and HPV-OPSCC in particular, in a representative sample of the German population.

**Materials and Methods:**

As part of an online, population-based survey, an electronic questionnaire was administered to a representative sample of 1,095 adult individuals with a specific emphasis on awareness of HPV, transmission, and indicator symptoms of oropharyngeal cancer. Statistical analysis of levels of awareness and relation of these to age, gender, and socioeconomic background were conducted using the IBM SPSS Statistics Version 25.0.

**Results:**

699/1,095 (63.8%) subjects had never heard of HPV. Of the subjects with awareness for HPV, 210 knew that HPV could be transmitted during sex (58.3%) and 138 recognized HPV as a risk factor for OPSCC (14.2%), unrelated to gender (*p* = 0.357), educational status (*p* = 0.581), or family status (*p* = 0.719). 416 subjects knew that a preventive vaccine against HPV existed (44.9%). Women were significantly more aware of HPV (34.2% vs. 22.8%, *p* < 0.001) and the vaccination (56.4% vs. 32.7%, *p* < 0.001) as were men. Younger individuals (age group 25–34) were significantly more aware of HPV (*p* < 0.001), likely as they were offered and/or had received the HPV vaccination. There was no regional variation of HPV awareness within the German state (*p* = 0.051).

**Conclusion:**

Here we demonstrate a significant lack of awareness of HPV and HPV vaccination in a representative sample of the German population. Levels of awareness of the link of HPV and oropharyngeal cancer are particularly low, bearing in mind that this cancer is commonly affecting men and incidence rates are rapidly rising in many European countries and the USA. Awareness programs and further education are required to tackle the low awareness rates and increase the uptake of the vaccination program not only in Germany, but also worldwide.

## Introduction

Human papillomaviruses (HPV) are widespread DNA viruses that can infect epithelial cells of the skin and mucosa and are the second most important infectious agent for cancer after *Helicobacter pylori* [1]. Most HPV infections remain clinically unapparent and clear spontaneously [2], but persisting HPV infections can cause a variety of benign and malignant lesions. Worldwide, the oncogenic or high-risk HPV types 16 and 18 contribute to more than 73% cases of all HPV-related cancers and the most commonly affected anatomic locations are the anogenital and head and neck region [1, 3]. HPV-attributable cancers in the head and neck region are most frequently located in the oropharynx (palatine tonsils and base of tongue), and increasing incidences are being noted worldwide. Changes in sexual behaviours in cohorts of individuals born during the 1930s–1950s are believed to be responsible for this increase [4], and a further rise amongst German and US seniors older than 65 years is anticipated by approximately 50% over the next decade [1, 4, 5, 6]. Unfortunately, there is an absence of reliable screening and secondary prevention strategies for oropharyngeal squamous cell carcinomas (OPSCC), and therefore, it seems that the prophylactic HPV vaccination has the greatest potential to prevent not only HPV-positive oropharyngeal cancers but also a variety of other high- and low-risk (LR) HPV-attributable diseases [7, 8, 9, 10]. Due to the high prophylactic efficacy, the nonavalent HPV vaccine (Gardasil 9®) has thus been approved by the FDA (Food and Drug Administration) for both, males and females [11, 12, 13].

Therefore, HPV vaccination has been recommended in standard vaccination programs in many countries since 2006/2007 for girls and female adolescents. The official recommendation for young males only followed in 2011 in the USA and 2018 in Germany [4]. HPV vaccination has the potential to decrease the burden of HPV-related disease amongst young HPV-unexposed adolescents, but unfortunately, there is a huge disparity between the rates desired by public health advocacy groups to achieve strong herd immunity and actual vaccination rates [14]. This has been shown for various countries, including the USA and Germany. In Germany, the rates of HPV vaccination in 2019 amongst young girls were only 47.2% overall with a notable regional difference between the western federal states (e.g., Bremen with 27.7%) in comparison to the eastern federal states (e.g., Saxony-Anhalt with 66.9%). The rate for the only recently recommended HPV vaccination for boys was much lower with 5.1% [15]. Considering the high numbers of HPV-attributable preventable diseases, rising incidence trends for HPV-related OPSCC and the relatively low HPV vaccination rates worldwide, it is important to understand the current status of HPV awareness in the general population.

The aim of this study was therefore to investigate the level of awareness for Head and Neck cancer, for HPV as a causative agent, and for HPV vaccinations in a cohort of German adult citizens with a specific emphasis on quantitative changes in awareness across birth cohorts, socioeconomic backgrounds, and gender. We also tried to comprehend the current level of knowledge on other types of risk factors for OPSCC and symptoms for Head and Neck cancer. This study contributes a large-scale dataset on HPV awareness in order to support the need for the reinforcement of a nationwide HPV vaccination program, particularly for young males.

## Materials and Methods

### Survey Participants

In this study, an electronic questionnaire was administered to a sample of 1,095 individuals representative of the German population via Dynata UK in line with advice from our statistics team and in line with our previously published work [16]. The study was conducted gradually in order to match age and gender with the aim of having a balanced study population. Matching of further criteria was planned but not feasible (e.g., stratification for educational status). The data collection was anonymized. Ethics approval for this study was not required, as it was an anonymous survey and did not include patients. This votum was granted by the Ethics Commission of the University Witten-Herdecke. The online questionnaire consisted of 42 questions assessing the interviewees' awareness on HPV, HPV vaccination, symptoms, and risk factors for oropharyngeal cancer. Information regarding sociodemographic characteristics as well as nicotine and alcohol abuse was also collected (shown in online suppl. File; for all online suppl. material, see www.karger.com/doi/10.1159/000525697).

### Statistical Analyses

In order to compare awareness amongst different characteristics of participants, χ^2^ tests were performed. For the analysis of correlations, a Spearman's rank correlation coefficient (Spearman's rho) was calculated. For all statistical analyses, SPSS software (IBM SPSS Statistics Version 25.0, IBM Corp., Armonk, NY) was used and a *p* value <0.05 was considered as significant.

## Results

### Demographics

Of the 1,095 subjects used for analysis (shown in Table 1), 546 were women (49.9%) and 549 were men (50.1%). Sixty-two subjects (5.7%) were between 18 and 24 years of age, and the other age categories were represented by 15.3% (35–44 years)–21.0% (55–64 years) of the participants. Most of the subjects were living in the western federal states of Germany (*n* = 878, 80.2%). More than half of the subjects (*n* = 590, 53.9%) were married or in a long-time relationship (*n* = 86, 7.9%). The rest of the subjects declared either a single status or were divorced (*n* = 419, 38.5%). 27.5% of the subjects stated that they did not have any school graduation and 36.5% (*n* = 400) stated that they had less than a high school graduation. Subjects with a degree (high school graduation, apprenticeship, and university studies) were underrepresented in this study (*n* = 74, 6.7%), even though 29.2% ticked “other” as the level of education. Almost two-thirds of the subjects were current smokers or smoked tobacco in the past (*n* = 298, 63.7%), and approximately one-third of the subjects were never-smoker (*n* = 393, 35.9%). More than two-thirds of the subjects drank at least one unit of alcohol per week (shown in Table 1).

### HPV Awareness

#### Overall HPV Awareness

Based on this survey, 278/1,095 (25.4%) subjects stated that they have heard of HPV. 699/1,095 (63.8%) have never heard of HPV, and the other subjects were either uncertain or did not reply to that question. Of the 278 subjects with HPV awareness, 37 (27.4%) stated that HPV is very rare. For further analysis, subjects who never heard of HPV or were uncertain were excluded.

### HPV Awareness and Gender

Women have heard of HPV more frequently than men: 166/485 (34.2%) women have heard of HPV and only 112/492 (22.8%) men have heard of HPV (*p* < 0.001).

### HPV Awareness and Age

The awareness for HPV is age dependent. Subjects in the age group 25–34 have heard of HPV more frequently (78/179, 43.6%) in comparison to the other age groups (e.g., age group 65–74 has the lowest awareness for HPV with only 12.8% [24/188]) (*p* < 0.001).

### HPV Awareness and Educational Qualification/Occupational Status

When comparing the educational qualification and the awareness for HPV, there is a significant correlation between the levels of qualification. Subjects who have not finished school education significantly knew more frequently about HPV (*n* = 82/280, 29.3%) than subjects who had a school leaving certificate (*n* = 86/407, 21.1%) (*p* = 0.050). Although there is a slight relation between age and educational qualification (χ^2^ test, *p* < 0.001), the correlation is only very small (Spearman's rho = −0.016), which means that the influence of educational qualification on HPV awareness cannot be explained by the influence of the age. Subjects who are retired knew less frequently about HPV (*n* = 37/270, 13.7%) than subjects who are actively working (*n* = 195/558, 34.9%) (*p* < 0.001).

### HPV and Regional Variation

There was no significant regional variation in HPV awareness observable. Inhabitants of the western federal states of Germany generally knew more often about HPV than the ones living in the eastern states (29.8% [*n* = 239/803] vs. 22.4% [*n* = 39/174]), but these results were not significant (*p* = 0.051).

### HPV Awareness and Family Status

There was no significant difference in HPV awareness between subjects who had children (*n* = 148/565, 26.2%) and subjects who did not have children (*n* = 130/412, 31.6%) (*p* = 0.067).

### HPV Vaccination Awareness

#### Overall HPV Vaccination Awareness

Based on this survey, 413/919 (44.9%) subjects stated that they have heard of an HPV vaccination. 506/919 (55.1%) have never heard of an HPV vaccination (again, the subjects who were uncertain or did not reply to that question were excluded for analysis).

### HPV Vaccination Awareness and Gender

Women have heard of an HPV vaccination significantly more frequent than men: 268/475 (56.4%) women have heard of an HPV vaccination in comparison to men with 32.7% (*n* = 145/444) (*p* < 0.001).

### HPV Vaccination Awareness and Age

The awareness for HPV vaccination is again age dependent. Subjects in the age group 25–34 have heard of an HPV vaccination significantly more frequent (*n* = 96/177, 54.2%) in comparison to the other age groups (e.g., age group 65–74 has the lowest awareness for HPV vaccination with only 33.1% [*n* = 55/166]) (*p* = 0.019).

### HPV Vaccination Awareness and Family Status

There was no significant difference in HPV vaccination awareness between subjects who had children (*n* = 248/530, 46.8%) and subjects who did not have children (*n* = 165/389, 42.4%) (*p* = 0.188).

### Risk Factors for and Symptoms of OPSCC

#### Awareness for Overall Risk Factors for OPSCC

As regards the awareness for overall risk factors for OPSCC, 404/1,095 (36.9%) subjects stated that excessive alcohol consumption can be a causative factor for OPSCC, 765/1,095 (69.9%) subjects stated that smoking and 280/1,095 (25.6%) subjects stated that poor oral hygiene is a causative agent. Only 181/1,095 (16.5%) subjects declared that HPV is a causative agent. At the same time, 188/1,095 (17.2%) stated that the herpes simplex virus is a causative agent.

### Awareness for HPV as a Risk Factor for OPSCC and Gender, Age, and Educational/Family Status

The majority of subjects stated that they are not aware of the fact that HPV could cause OPSCC as well as cervical cancer (*n* = 836/974, 85.8%) and only 14.2% (*n* = 138/974) are aware. Yet, the awareness for HPV as a causative agent for OPSCC was significantly higher in the age group 25–34 with 26.7% (*n* = 47/176) and lowest in the age group 65–74 with 4% (*n* = 7/175) (*p* < 0.001). The awareness was not related to gender (*p* = 0.357), educational status (*p* = 0.581), or family status (*p* = 0.719).

### Correlation between HPV Awareness and Symptoms/Transmission for OPSCC

Overall, only a slight positive tendency of correlation can be seen between subjects, who have heard of HPV and the awareness of possible symptoms for OPSCC (Spearman's rho ranging from 0.074 [loss of teeth] to 0.199 [swelling of the throat], shown in Table 2). The subjects who have heard of HPV answered that HPV can be transmitted by sexual intercourse (58.3%, *n* = 210/360) or by oral sex (53.8%, *n* = 164/305). Almost 1/5 of the subjects who have heard of HPV stated that HPV can be the cause for HIV/AIDS (19.4% [*n* = 54/278]).

## Discussion

The results of our study demonstrate that there is a significant lack of HPV awareness in a representative cohort of German citizens. Most disturbingly, there is a very limited knowledge of HPV as a causative agent for OPSCC. Almost two-thirds of the subjects have never heard of HPV and only 14.2% were aware that HPV could cause OPSCC. These findings are in line with the current literature, which shows that awareness for HPV as a causative agent for OPSCC on an international level is low. A recent review analysed 32 studies and showed that there is a knowledge gap of HPV-associated OPSCC, not only for the general population but also for health care providers [17, 18]. The proportion of the general population and health care providers with knowledge of HPV ranged from 16% to 75% and 21% to 84%, respectively. Knowledge of HPV-associated OPSCC was greater in health care providers and ranged from 22% to 100% compared with the general population, which ranged only from 7% to 57% [17], but in general it was still low. Therefore, not only the lack of HPV awareness in the general population seems to be a risk but also the lack in health care providers.

Furthermore, the awareness for HPV and HPV as a causative agent for cancer was significantly age dependent and younger subjects had a higher awareness, but still, only 43.6% of the 25- to 34-year olds have heard of HPV and only 26.7% of the same age group have heard of HPV as a causative agent for cancer. Comparatively, only 12.8% of the 65- to 74-year olds had an awareness for HPV and only 4% knew about the fact that HPV could cause OPSCC. This is in line with the finding of another study, in which people older than 65 years had heard of HPV significantly less than the younger generations (*p* = 0.001) [16]. Still, levels of HPV and HPV-OPSCC awareness as well as HPV vaccination in young populations are alarmingly low [19, 20]. In an online survey on head and neck cancer, which was conducted amongst 1,903 subjects aged 18–35 years in Poland, the overall awareness for HPV as a causative agent for head and neck cancer was only 37.2% [20]. Similarly, public awareness of HPV in a Dutch cohort with 1,044 participants was of only 30.7% and awareness of the HPV vaccine was only 49.7% [16].

We were also able to show significantly higher awareness in German women on both, HPV and HPV vaccination (34.2% and 56.4%) (age dependent), and it does not come surprising that there is a disparity in knowledge and awareness of HPV vaccines between men and women [21, 22]. Several studies have shown that women are much more informed about HPV vaccines and a recent study has shown that women were 225% more likely to have heard of HPV and 281% more likely to have heard of the HPV vaccine [22]. Nonetheless, another study has shown that the awareness for eligibility of the HPV vaccine for both sexes is very low [23]. In addition, there was no regional variation of HPV awareness and no difference of HPV awareness when comparing the family status. For instance, subjects with children did not have a higher HPV awareness than the rest of the interviewees. A recent study showed that HPV-related cancer survivors had a higher awareness for HPV, thought of the HPV vaccine as safe, and were more likely to vaccinate their children or recommend the vaccine, respectively. These survivors, if educated and empowered with information about the efficacy of HPV vaccination, vaccination recommendations, and vaccine safety, could serve as a powerful tool of information diffusion [24].

Nonetheless, there are clear limitations of this study, and even though the number of subjects is quite high, the study cohort was too small to stratify for more than age and gender. Stratification by educational status and region was not possible. Furthermore, we did not include questions on how the subjects were informed about HPV making it difficult to understand, which would be the best way to address the lack of HPV knowledge.

Apart from the extreme health burden that HPV can cause in the different fields of medicine with a massive impact on overall survival, there is a huge economic burden, also of HPV-related OPSCC worldwide and in Germany [25, 26, 27, 28, 29]. These costs incur by hospitalization with treatment (medical services covering physician visits and diagnostics, therapy), outpatient management, outpatient chemotherapy, long-term care, premature retirement, and premature death [26]. Reaching high numbers for HPV awareness therefore should be the goal not only for health care providers but also for the general population and the health care system. The aim needs to be to enlarge the HPV advocacy platform, not only in Germany but worldwide.

## Conclusion

Our data demonstrate a significant lack of HPV awareness in a representative cohort of German adult citizens. Particularly, HPV as a causative agent for OPSCC is commonly unknown. Sociodemographic and behavioural factors are less likely to be the reason for this awareness. Instead, a lack of health education has most likely contributed to the alarming data, indicating the need for further education.

## Statement of Ethics

Ethics approval was not required as it was an anonymous survey and did not include patients (Ethics Committee of the University Witten-Herdecke). Informed consent to participate was not directly obtained but inferred by completion of the questionnaire.

## Conflict of Interest Statement

The authors have no conflicts of interest to declare.

## Funding Sources

No funding was obtained.

## Author Contributions

Shachi Jenny Sharma and Matt Lechner: study design, acquisition, analysis, interpretation, drafting, accountability, and final approval. Volker H. Schartinger, Lisa Collin, Liam Sutton, and David Riedl: study design, acquisition, drafting, accountability, and final approval. Nora Wuerdemann and Christine Langer: interpretation, drafting, accountability, and final approval. Kathrin Moellenhoff: analysis, interpretation, drafting, accountability, and final approval. Alexander Kreuter: interpretation, drafting, accountability, and final approval. Ulrike Wieland: analysis, interpretation, drafting, accountability, and final approval. Jens Peter Klussmann: study design, acquisition, analysis, interpretation, accountability, and final approval.

## Data Availability Statement

All data generated or analysed during this study are included in this article. Further enquiries can be directed to the corresponding author.

## Supplementary Material

Supplementary data

## Figures and Tables

**Table 1:** Demographic characteristics of the sample

	*N*	%
Age		
<17	3	0.3
18–24	62	5.7
25–34	193	17.6
35–44	168	15.3
45–54	198	18.1
55–64	230	21.0
65–74	210	19.2
≥75	30	2.7
Gender		
Male	549	50.1
Female	546	49.9
Marital status		
Single	286	26.1
Relationship >1 year	86	7.9
Married	590	53.9
Separated or divorced	133	12.1
Area of residence		
Old states of Germany	878	80.2
New states of Germany	194	17.7
Level of education		
No school graduation	301	27.5
Less than high school graduation	400	36.5
High school graduation	7	0.6
Apprenticeship	57	5.2
University	10	0.9
Other	320	29.2
Smoking status		
Current smoker or chewing tobacco	47	40.8
Ex-smoker or previous tobacco chewing	251	22.9
Never smoked/chewed tobacco	393	35.9
Alcohol intake, units/week		
Never drink alcohol	396	36.2
1–14	577	52.7
15–21	75	6.8
>21	26	2.4

Sample size: *n* = 1,095.

**Table 2:** Correlation (Spearman's rho) between HPV awareness and symptoms for OPSCC

	HPV awareness
Swelling of the neck	0.175
Bleeding from the mouth/throat	0.146
Swelling of the throat	0.199
Sore throat	0.079
Earache	0.131
Loss of appetite	0.127
Headache	0.099
Loss of teeth	0.074
Swallowing pain	0.144
Scratching/foreign body sensation of the throat	0.141
