# Supplementary material for: Awareness of Human Papillomavirus (HPV) and HPV Vaccination amongst the General Population in Germany: Lack of Awareness and Need for Action
Source: Oncol Res Treat. 2022 Jul 18;45(10):561–7. doi: 10.1159/000525697 (PMC9677834; doi:10.1159/000525697)
Supplement: Supplementary file 1 — Supplementary data [file ort-0045-0561-s01.docx]

**Fragebogen über zwei unterschiedliche Erkrankungen des Rachens**

Sehr geehrte Damen und Herren!

Vielen Dank für Ihr Interesse an dieser Studie und für Ihre Zeit, die das Ausfüllen des Fragebogens benötigt. Der Fragebogen besteht aus 3 Teilen. Zunächst werden Sie gebeten ein paar allgemeine Fragen zu Ihrer Person zu machen. Anschließend werden in zwei getrennten Kapitel Fragen zum Rachen/Mandelkrebs (Oropharynxkarzinom) und zum Schnarchen gestellt.

Wir bitten wir Sie, diesen Fragebogen auszufüllen und uns damit wertvolle Informationen zu liefern.

- Bitte beantworten Sie die Fragen, indem Sie sich nur auf Ihre **derzeitige, aktuelle Situation** beziehen, darauf wie es Ihnen zur Zeit geht bzw. wie Sie Ihre Situation zur Zeit erleben
- Bitte kennzeichnen Sie den Fragebogen **nicht** namentlich. Die Fragebögen werden anonymisiert und vertraulich behandelt. Die erhobenen Daten unterliegen dem Datenschutz.
- Bitte füllen Sie den Fragebogen möglichst **vollständig** aus.
- Kreuzen Sie bitte auf jeder Fragebogenseite diejenige Antwort an, die am besten auf Sie zutrifft.
- Wenn keine der Antworten voll auf Sie zutrifft, dann kreuzen Sie bitte diejenigen an, die am ehesten auf Sie zutrifft.
- Bitte denken Sie nicht lange über Ihre Antwort nach!
- Es gibt keine richtigen oder falschen Antworten.
- Beantworten Sie bitte alle Fragen ehrlich!

Herzlichen Dank für Ihre wertvolle Mitarbeit!

**TEIL 1**

**Zunächst ein paar Fragen zur Ihrer Person:**

1. **Wie alt sind Sie? ________**Jahre
2. **Sind Sie**

| - - männlich | - - weiblich |
| --- | --- |

1. **Wie ist Ihr aktueller Familienstand?**

| - - alleinstehend | - - geschieden/getrennt |
| --- | --- |
| - - verheiratet/Lebensgemeinschaft | - - feste Partnerschaft (> 1 Jahr) |

1. **Wie ist Ihre aktuelle Wohnsituation**

| - - alleinlebend | - - lebe bei der Herkunftsfamilie |
| --- | --- |
| - - lebe mit Partner/eigener Familie/Kindern | - - lebe in Wohngemeinschaft |
| - - lebe in Institution |  |

1. **Was ist Ihre höchste abgeschlossene Ausbildung?**

| - - Schule noch nicht abgeschlossen | - - Abitur oder berufsbildende höhere Schule |
| --- | --- |
| - - kein Schulabschluss | - - Universität |
| - - Pflichtschulabschluss ohne Lehre | - - sonstiges/unbekannt |
| - - Pflichtschulabschluss mit Lehre |  |

1. **Frage zu Ihrer Berufstätigkeit?**

| - - noch in Ausbildung | - - pensioniert |
| --- | --- |
| - - Hausmann / Hausfrau | - - Pension befristet / laufender Antrag |
| - - Arbeitslos | - - Krankenstand (länger als drei Monate) |
| - - teilzeitbeschäftigt | - - Sonstiges |
| - - voll erwerbstätig |  |

1. **Haben Sie Kinder?**

- Nein
- Ja 🡪 Anzahl der Kinder: ______

Alter ältestes Kind: ______ Jahre

Alter jüngstes Kind: ______ Jahre

1. **Rauchen Sie oder kauen Sie Tabak?**
   - Raucher oder Tabak-Kauer
   - Ex-Raucher oder früher Tabak-Kauer
   - Nie geraucht/Nie Tabak gekaut

Wenn ja, wieviele Zigaretten täglich

- - ≤20
  - 21 bis 35
  - >35

Wenn ja, wie lange? ___________ Jahre

1. **Wie viele Einheiten Alkohol trinken Sie durchschnittlich pro Woche?**
   - Ich trinke keinen Alkohol
   - 1-14 Einheiten (ca. 0,25 – 3,5 l Bier / 1 – 14 kleine Gläser Wein)
   - 15-21 Einheiten (ca. 3,75 – 5,25 l Bier / 15 – 21 kleine Gläser Wein)
   - Über 21 Einheiten (mehr als 5,25 l Bier / mehr als 21 Gläser Wein)
2. **Trinken Sie mehr als 4 alkoholische Getränke/Einheiten pro Tag?**

(1 Einheit ist ca. 0,25 l Bier, ein kleines Glas Wein oder 1 Schnaps)

- - Ja
  - Nein

1. **Wo leben Sie?**

| - - Baden-Württemberg | - - Bayern | - - Berlin |
| --- | --- | --- |
| - - Brandenburg | - - Bremen | - - Hamburg |
| - - Hessen | - - Mecklenburg-   Vorpommern | - - Niedersachsen |
| - - Nordrhein-Westfalen | - - Rheinland-Pfalz | - - Saarland |
| - - Sachsen | - - Sachsen-Anhalt | - - Schleswig-Holstein |
| - - Thüringen |  |  |

1. **Ihre Staatsbürgerschaft:**
   - Deutsch
   - Nicht-Deutsch
2. **Wo sind Sie geboren?**
   - In Deutschland
   - In einem anderen Land, und zwar: ______________________

**TEIL 2**

**Fragebogen zum Rachen-/Mandelkrebs (Oropharynxkarzinom):**

Das Ziel dieser Studie ist es, das Wissen über Rachen-/Mandelkrebs in der Bevölkerung einzuschätzen. Vom Ergebnis dieser Studie hängt es ab ob, eine öffentliche Sensibilisierungskampagne zur Steigerung des Bewusstseins in der Bevölkerung nötig ist. Der Rachenkrebs oder Mandelkrebs ist auch bekannt als das Oropharynxkarzinom.

1. **Was sind Warnhinweise und Symptome des Rachenkrebs? Wenn Sie unsicher sind, bitte kreuzen Sie „Weiß nicht“ an.**

|  | Ja | Nein | Weiß nicht |
| --- | --- | --- | --- |
| Schwellung außen am Hals | ⃝ | ⃝ | ⃝ |
| Blutung aus Mund oder Rachen | ⃝ | ⃝ | ⃝ |
| Schwellung oder Gewächs im Rachen | ⃝ | ⃝ | ⃝ |
| Halsschmerzen | ⃝ | ⃝ | ⃝ |
| Ohrenschmerzen | ⃝ | ⃝ | ⃝ |
| Appetitverlust | ⃝ | ⃝ | ⃝ |
| Kopfschmerzen | ⃝ | ⃝ | ⃝ |
| Zahnverlust | ⃝ | ⃝ | ⃝ |
| Schmerzen beim Schlucken | ⃝ | ⃝ | ⃝ |
| Kratzen im Hals/Fremdkörpergefühl | ⃝ | ⃝ | ⃝ |

1. **Was sind die häufigsten Risikofaktoren für Rachenkrebs?**

|  | Ja | Nein | Weiß nicht |
| --- | --- | --- | --- |
| Exzessiver Alkoholkonsum | ⃝ | ⃝ | ⃝ |
| Tabak rauchen | ⃝ | ⃝ | ⃝ |
| Tabak kauen | ⃝ | ⃝ | ⃝ |
| Kauen von Betel-Blättern | ⃝ | ⃝ | ⃝ |
| Kauen von Catchu und Arecanuss | ⃝ | ⃝ | ⃝ |
| Marijuana Konsum | ⃝ | ⃝ | ⃝ |
| Herpes simplex Virus Infektion | ⃝ | ⃝ | ⃝ |
| Humanes Papillomavirus (HPV) Infektion | ⃝ | ⃝ | ⃝ |
| Krebsfälle in der Familie | ⃝ | ⃝ | ⃝ |
| Konsum von Obst und Gemüse | ⃝ | ⃝ | ⃝ |
| Sonnenexposition | ⃝ | ⃝ | ⃝ |
| Mangelnde Mundhygiene | ⃝ | ⃝ | ⃝ |

1. **Haben Sie vor dem Ausfüllen dieses Fragebogens schon einmal etwas von HPV (Humanes Papillomavirus) gehört?**
   - Ja
   - Nein
   - Nicht sicher
2. **Wussten Sie, dass HPV, welches auch ursächlich für Gebärmutterhalskrebs ist, auch ein Risikofaktor für das Rachenkrebs ist?**
   - Ja
   - Nein
   - Ich habe noch nie von HPV gehört
3. **Bitte lesen Sie die folgenden Aussagen und kreuzen Sie an, ob diese richtig oder falsch sind**

|  | richtig | falsch | Nicht sicher |
| --- | --- | --- | --- |
| a) HPV kann durch Geschlechtsverkehr übertragen werden | ⃝ | ⃝ | ⃝ |
| b) HPV kann die Ursache für HIV/AIDS sein | ⃝ | ⃝ | ⃝ |
| c) HPV ist sehr selten | ⃝ | ⃝ | ⃝ |
| d) HPV kann durch Oralverkehr übertragen werden | ⃝ | ⃝ | ⃝ |

1. **Wussten Sie, dass es eine Impfung gegen HPV (Humanes Papillomavirus) gibt?**
   - Ja
   - Nein
   - Ich habe noch nie von HPV gehört

**TEIL 3**

**Schlaf-Fragebogen**

Das Ziel dieser kurzen Umfrage ist es, die Häufigkeit von Schnarchen und Atempausen (und die assoziierten Erkrankungen) der deutschen Bevölkerung zu evaluieren und Gemeinsamkeiten zwischen diesen Erkrankungen und sozialdemographischen Variablen zu finden (andere gesundheitliche Probleme, Verkehrsunfälle und Bedarf medizinischer Einrichtungen)

**1. Schnarchen Sie während des Schlafes? Hat Ihr Partner Ihnen gesagt, dass Sie in der Nacht schnarchen?**

- - Ja (🡪 weiter zur Frage 1a)
  - Nein (🡪 weiter zur Frage 2)

**1.a. Wie oft**

| - - nie / selten | - - 1 – 2 Nächte / Woche | - - 3 Nächte / Woche |
| --- | --- | --- |
| - - 4 – 5 Nächte / Woche | - - 6 – 7 Nächte / Woche |  |

**2. Schläft Ihr Partner aufgrund des Schnarchens in einem anderem Raum?**

- - ja
  - nein
  - Ich lebe mit keinem Partner zusammen

**3. Wurde Ihnen gesagt, dass Sie während des Schlafens für kurze Zeit zu Atmen aufhören (Atempausen) oder wachen Sie um Luft ringend in der Nacht auf?**

- - Ja (🡪 weiter mit Frage 3a)
  - Nein (🡪 weiter mit Frage 4)

**3.a. Wie oft pro Woche hören Sie während Ihres Schlafes für kurze Zeit auf zu atmen (Atempausen) bzw. wachen um Luft ringend auf?**

| - - nie / selten | - - 1 – 2 Nächte / Woche | - - 3 Nächte / Woche |
| --- | --- | --- |
| - - 4 – 5 Nächte / Woche | - - 6 – 7 Nächte / Woche |  |

**4. Wie groß sind Sie (Körpergröße in [cm])? …………….. cm**

**5. Wie schwer sind Sie (Körpergewicht in [kg])? …………….. kg**

**-> BMI wird vom System berechnet**

**6. Wie würden Sie Ihre Körperform am ehesten beschreiben?**


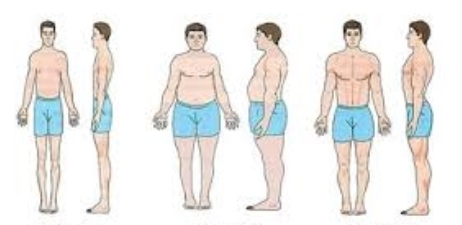


b)

c)

a)

**7. Messen Sie bitte mit Hilfe eines Maßbandes und erfassen Sie folgende Messungen**

a) Ihren Kopfumfang: …………..cm ⃝ nicht durchgeführt

b) Ihren Halsumfang: …………..cm ⃝ nicht durchgeführt

c) Ihren Brustumfang: …………..cm ⃝ nicht durchgeführt

d) Ihren Bauchumfang: …………..cm ⃝ nicht durchgeführt

**8. Wählen Sie eine der unten gezeigten Möglichkeiten aus, die am ehesten die Position Ihres Oberkiefers gegenüber Ihrem Unterkiefer beschreibt.**


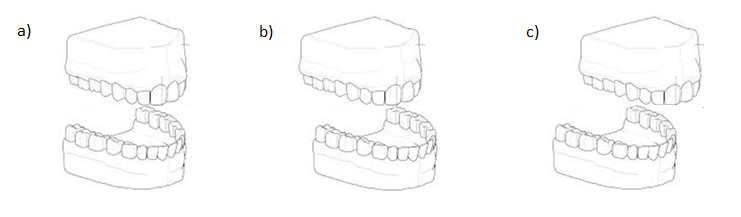


**9. Wie würden Sie Ihren Schlaf beschreiben?**

- - zu kurz
  - zu lang
  - genau richtig

|  | Nie / selten | 1-2 Tage / Woche | 3 Tage / Woche | 4-5 Tage / Woche | 6-7 Tage / Woche |
| --- | --- | --- | --- | --- | --- |
| **10. Fühlen Sie sich erholt, wenn Sie aufwachen?** | **⃝** | **⃝** | **⃝** | **⃝** | **⃝** |
| **11. Wie oft wachen Sie in der Nacht auf?** | **⃝** | **⃝** | **⃝** | **⃝** | **⃝** |
| **12. Wie oft haben Sie Schwierigkeiten einzuschlafen?** | **⃝** | **⃝** | **⃝** | **⃝** | **⃝** |
| **13. Wie oft wachen Sie in der Früh zu zeitig auf und können dann nicht mehr einschlafen?** | **⃝** | **⃝** | **⃝** | **⃝** | **⃝** |

**15. Fühlen Sie sich tagsüber müde oder schläfrig?**

- - Ja (🡪 weiter zu Frage 15a)
  - Nein (🡪 weiter zu Frage 16)

**15a. Wie oft?**

| - - nie / selten | - - 1 – 2 Nächte / Woche | - - 3 Nächte / Woche |
| --- | --- | --- |
| - - 4 – 5 Nächte / Woche | - - 6 – 7 Nächte / Woche |  |

**15.b. Wie stark ist Ihre Müdigkeit/Schläfrigkeit?**

- - gering
  - mittelgradig
  - schwer

**16. Wie oft machen Sie tagsüber ein Nickerchen?**

| - - nie / selten | - - 1 – 2 Nächte / Woche | - - 3 Nächte / Woche |
| --- | --- | --- |
| - - 4 – 5 Nächte / Woche | - - 6 – 7 Nächte / Woche |  |

**17. Leiden Sie unter Alpträumen?**

- - nie
  - einmal im Monat
  - mehr als einmal im Monat

**18. Wieviel Kaffee oder Tee trinken Sie am Tag**

- - 0 Tassen
  - 1-3 Tassen
  - 3-5 Tassen
  - 6 oder mehr Tassen

|  | JA | NEIN |
| --- | --- | --- |
| **19. Leiden Sie an einer Obstuktion der Atemwege, wie Asthma oder COPD?** | ⃝ | ⃝ |
| **20. Haben Sie eine Funktionsstörung der Schilddrüse** | ⃝ | ⃝ |
| **21. Leiden Sie an Diabetes (Zuckerkrankheit)** | ⃝ | ⃝ |
| **22. Haben Sie einen zu hohen Blutdruck?** | ⃝ | ⃝ |
| **23. Leiden oder litten Sie an Blutarmut (Anämie)** | ⃝ | ⃝ |

**24. Was hat Ihr Hausarzt zuletzt über Ihren Blutdruck gesagt?**

- - mein Blutdruck war zu niedrig
  - mein Blutdruck war zu hoch
  - mein Blutdruck war in Ordnung
  - ich kann mich nicht erinnern
  - ich war seit mehr als einem Jahr nicht beim Hausarzt

|  | Nie / selten | 1-2 Tage / Woche | 3 Tage / Woche | 4-5 Tage / Woche | 6-7 Tage / Woche |
| --- | --- | --- | --- | --- | --- |
| **25. Wie oft nehmen Sie Medikamente, um Ängste zu bewältigen?** | **⃝** | **⃝** | **⃝** | **⃝** | **⃝** |
| **26. Wie oft nehmen Sie Medikamente gegen Depressionen?** | **⃝** | **⃝** | **⃝** | **⃝** | **⃝** |
| **27. Wie oft nehmen Sie Medikamente um besser einzuschlafen?** | **⃝** | **⃝** | **⃝** | **⃝** | **⃝** |

**28. Sind Sie während des letzten Jahres einmal am Steuer Ihres Fahrzeuges eingeschlafen?**

- - ja, ein- oder zweimal
  - ja, mehrere Male
  - nie

**29. Hatten Sie im vergangenen Jahr einen Verkehrsunfall?**

- - ja
  - nein
  - nicht zutreffend

**30. Haben Sie im letzten Jahr selbständig einen Arzt wegen dem Schnarchen oder wegen der Atempausen aufgesucht?**

- - ja
  - nein
  - nicht zutreffend

**31. Gibt es in Ihrer Familie (Vater, Mutter oder Geschwister) jemanden, der an einem Schlafapnoesyndrom leidet?**

- - ja
  - nein
  - ich weiß es nicht

Vielen Dank für das Ausfüllen dieses Fragebogens.
